# Albuminuria and Life Assurance

**Published:** 1894-11-17

**Authors:** 


The Hospital

An Institutional Journal of

The Medical Sciences and Hospital Administration, with

Vol. XVII.—No. 425.] AN EXTRA NURSING SUPPLEMENT. [Nov. 17, 1894.

### ALBUMINURIA AND LIFE ASSURANCE.

From a medical, as well as from a commercial, point of view, life assurance is a much less simple proceeding than it was to the medical examiner of thirty or forty years ago. In those times, so scientifically remote, large numbers of cases were accepted which would now be promptly rejected, and conversely a number, perhaps almost equally large, were rejected which would now be accepted. Albuminuria was then a word of import always fatal. It was held to be, of itself, pathognomonic of Bright's disease. In any case for examination, no matter at what hour of the day or under what circumstances, if albumin were found in the urine the medical examiner considered that he had but one duty to perform, and that was to reject the case absolutely. It is probable that even to-day the majority of examiners, not excepting some assurance specialists themselves, carry on their daily business on these principles.

Is this decision scientifically sound? Is it based upon data justified by physiology, pathology, and experience? We think it is not. If it be not, it would be well that medical examiners for life assurance should revise their conclusions in this regard. Some of them are already doing so, and it is certain that the ever increasing demands of the assurance societies for new business will induce them to encourage all those courageous and highly-skilled medical examiners who by a more competently scientific selection of lives can multiply assurers without increasing risks.

The real question for medical examiners, and, of course, for all other practitioners, to ask themselves is this: What is the actual vital significance of albuminuria, of albuminuria, that is, taken absolutely by itself? The significance of albumin, with certain varieties of tube casts in the urine, of albumin which is persistent, of albumin which is always or almost always present in large quantities, we need not here inquire further about; it is no doubt pathological, and practically always pathological. But when albumin is only occasionally present, present in small quantities, and never associated with tube casts of any kind, is it certain that in these cases it is always and invariably of true pathological import? We contend that it is not; and in this contention we claim the support of a rational physiology and also of competent medical authority.

In any case of albuminuria pure and simple, it is probable that the glomerulus of the kidney is the one and only source of the albumin. There have not been wanting careful observers, for example Von Wittich and Ludwig, who have contended that the simple transudation of albumin into Bowman's capsule is a purely physiological process, and that albumin is often so transuded without ever appearing in the urine at all, being reabsorbed by the epithelium of the tubules. The fact, however, that the epithelium of the tubules is secreting and not absorbent epithelium would appear sufficient to disprove that contention. But even though we may deny that the transudation of albumin is a daily and regular physiological phenomen, we need not deny that it is a fact of common occurrence. Moreover we can hardly contend with philosophic warrant that it is always a pathological phenomenon. Under certain circumstances of excessive exertion and high renal blood pressure, many persons will show the symptom of albuminuria who never show it at any other time. Is this physiological or is it pathological? Is not this the true state of the case, that the dividing line between the physiological and the pathological is here so very fine that none of us can be always certain when that line is crossed? The probability is that the line is often temporarily both crossed and recrossed without either the person himself or his physician ever becoming cognizant of the fact. It ought to be remembered too in this connection that just as theremay be infinite diversities of external feature and of internal histological structure in all sorts of persons who have nevertheless this circumstance in common that they are all in perfect health, so there may be very marked differences in the permeability of their blood vessels, glomerular coils included, without any real departure from the healthy standard. All these facts and circumstances require to be duly considered.

Experiments, sufficient in number to justify the generalisations made, have been carried out by many different renal investigators, which have warranted the conclusion that many persons show the symptom of uncomplicated albuminuria who have never had and are never likely to suffer from any of the many forms of Bright's disease. Sir Thomas Grainger Stewart has published a record of more than 500 cases of presumably healthy individuals of whom as many as 32·8 per cent. showed occasional albuminuria. Dr. Stirling examined an almost equal number, and found a still higher percentage. The experiences of Moxon and Pavy more or less coincided with these results. It has been found that many soldiers show albumin in their urine after a fatiguing day's march who never show it at any other time. Even so simple a form of exertion as long standing in the erect posture is sufficient to induce the symptom in some persons.

What is needed at the present time is a sound working generalisation, and thoroughly trustworthy methods of examining the urine. Dr. Saundby has given the profession a generalisation, which, with a little modification and widening in its application, would seem to be worthy of confidence. He lays down this rule, that in a case of albuminuria, if "the urine is normal in every other respect, if there be no tube casts, if the amount of solid matter excreted be sufficient, if there are no signs of cardiac hypertrophy or of high arterial tension, no retinal changes and no œdema," such a life "may be safely accepted by life assurance companies." This is a generalisation which obviously can only be applied in practice with very great discrimination. We should be disposed to add to it that not one examination should be sufficient, nor even two or three. At least twelve examinations of the urine should be insisted upon at considerable intervals. If it be objected that a good many medical men are not skilled examiners of the urine to the extent here demanded, we may remind our professional brethren that that difficulty need no longer stand in the way of their acceptance of responsibility, since the Clinical Research Association, for a very small fee, makes thoroughly trustworthy examinations for any of its members. In presenting these facts and arguments to professional readers, we have no desire to dogmatise. The subject is admittedly difficult, and good reasons can be advanced in favour of less liberal views than ours. This much, however, we may assert without fear of contradiction, that occasional and uncomplicated albuminuria is a very different thing from Bright's disease, and demands reconsideration at our hands in the interests of the public and the profession alike.

